# Segregation Engineering in MgO Nanoparticle-Derived Ceramics: The Impact of Calcium and Barium Admixtures on the Microstructure and Light Emission Properties

**DOI:** 10.1021/acsami.1c02931

**Published:** 2021-05-19

**Authors:** Thomas Schwab, Korbinian Aicher, Hasan Razouq, Gregor A. Zickler, Oliver Diwald

**Affiliations:** Department of Chemistry and Physics of Materials, Paris-Lodron University of Salzburg, Jakob-Haringer-Straße 2a, Salzburg 5020, Austria

**Keywords:** nanostructured ceramics, intergranular region, segregation engineering, ion segregation, inorganic phosphors, grain growth, photoemission properties

## Abstract

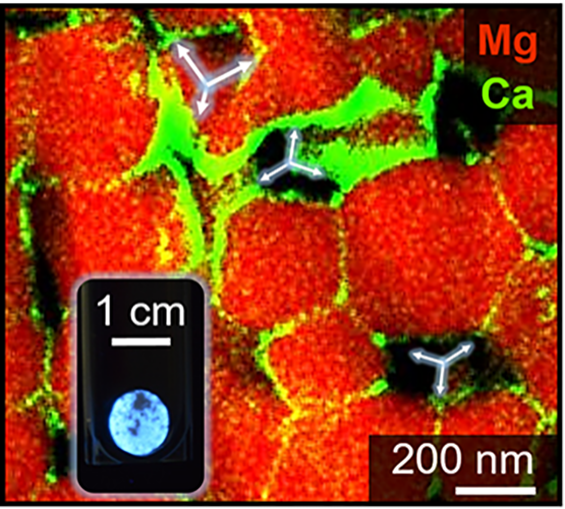

Nanostructured segregates of alkaline earth oxides exhibit bright photoluminescence emission and great potential as components of earth-abundant inorganic phosphors. We evaluated segregation engineering of Ca^2+^- and Ba^2+^-admixtures in sintered MgO nanocube-derived compacts. Compaction and sintering transform the nanoparticle agglomerates into ceramics with residual porosities of *Φ* = 24–28%. Size mismatch drives admixture segregation into the intergranular region, where they form thin metal oxide films and inclusions decorating grain boundaries and pores. An important trend in the median grain size evolution of the sintered bodies with *d*_Ca(10 at. %)_ = 90 nm < *d*_Ba(1 at. %)_ = 160 nm < ***d***_**MgO**_ = **250 nm** ∼ *d*_Ca(1 at. %)_ = 280 nm < *d*_Ba(10 at. %)_ = 870 nm is rationalized by segregation and interface energies, barriers for ion diffusion, admixture concentration, and the increasing surface basicity of the grains during processing. We outline the potential of admixtures on interface engineering in MgO nanocrystal-derived ceramics and demonstrate that in the sintered compacts, the photoluminescence emission originating from the grain surfaces is retained. Interior parts of the ceramic, which are accessible to molecules from the gas phase, contribute with oxygen partial pressure-dependent intensities to light emission.

## 1 Introduction

Internal interfaces and boundary regions between nanocrystalline grains have a key influence on the physico-chemical material properties. Hence, their design and formation are critical for the optimization of the structural and functional performance of sintered structures.^1−4^ Grain boundary (GB) engineering in combination with ion exsolution and strain effects has successfully been employed to achieve improved electrode materials.^5^ Another example for related development of functional materials is the adjustment of nanostructured SrTiO_3_ thermoelectrics via the addition of extrinsic elements into the GB phase together with control over oxygen partial pressure and temperature.^6^ The enhanced solubility and diffusion of transition-metal cations in and through GBs inside acceptor-doped CeO_2_, as the third example, were found to have a significant impact on the performance and durability of ceria-containing solid-state electrolytes.^7−10^

On alkaline earth metal oxides (AEOs) doped with different cationic admixtures, the study of interface modification utilizing admixture segregation has previously been performed on micro- and single crystalline materials for multiple reasons: first, the fundamental investigation of bulk and interface phenomena benefit from its simple structure and the pronounced ionicity of AEOs.^11−17^ Second, research in heterogeneous catalysis has revealed a number of key reactions where perfectly dissolved impurity ions and surface segregates act as active surface sites of the catalyst particles.^15,18−22^ Magnetism, which arises from the incorporation and interface segregation of transition-metal ions, has great potential for applications in spintronics and represents another example for functional properties that emerge from engineering the solid–solid interface.^23−25^

Apart from scintillation and dosimeter properties that were reported for mixed AEOs,^26,27^ their composite surface and interface structures exhibit promising optical properties. As demonstrated in previous studies, MgO nanocubes represent a suitable support material for isovalent Ca^2+^, Sr^2+^, and Ba^2+^ admixtures that – after impurity trapping inside the host particles during the combustion synthesis – undergo annealing-induced surface segregation to generate a new class of nanoparticle-based inorganic phosphors with bright photoluminescence (PL) emission in the range of visible light.^28−30^ Combined experimental and theoretical work has revealed that the photoexcitation and radiative deactivation processes involve surface Ca^2+^- and Ba^2+^-ions and sensitively depend on their dispersion and coordination state at the grain surface.^30^

Related understanding was obtained on non-consolidated powders of nanoparticles with free particle surfaces. Here, we have addressed for the first time and in the context of ceramics production two key questions: (i) how do these admixture segregates affect coarsening of nanometer-sized grains during microstructure evolution and sintering upon annealing of nanoparticle-derived compacts and (ii) can the optical properties that are specific to the free grain surfaces inside powders be preserved during ceramic manufacturing. Motivated by the link between the starting material properties, that is, size and composition distribution of the nanocrystals, conditions of material processing, microstructure evolution, and functional properties, we have performed this segregation and processing study on powders of extremely well-defined nanoparticles that were grown by flame spray pyrolysis (FSP). After material densification, the detailed structure characterization work with high-resolution transmission electron microscopy (HR-TEM) in combination with elemental mapping has aimed at the identification of emerging interfaces and phases, residual porosities and grain size distributions, as well as assessment of the elemental distribution of Ca^2+^- and Ba^2+^-admixtures over grains and grain interfaces. In the past, the effect of dopants on the energies of GBs and interfaces has been described for different ceramic systems.^3,31−36^ Decrease or increase of the interface energies affects the sintering behavior of the nanocrystalline ceramic and provides a handle to adjust the annealing-induced grain growth. As a case study, this work offers knowledge for precise control over admixture distribution between grains and their interfaces and, thus, on grain growth during sintering.

Knowledge about interfacial segregation in alloyed metal nanoparticles has been transferred to more complex systems such as doped binary and multinary metal oxides only recently. Together with the advance of new experimental methodologies and with support from modeling, interface engineering via solute segregation bears huge potential for the development of high-performance ceramics.^1,2,4,11,12,37−39^

## 2 Experimental Section

### 2.1 Flame Spray Pyrolysis

The flame spray reactor consists of two major parts, a burner and a powder collection unit. The liquid fuel-precursor mixture is supplied by a syringe pump (LA-160, HLL Landgraf) at a constant flow rate of 2.0 mL min^–1^ through a capillary of a binary spray nozzle. At the spray nozzle exit, the liquid feed is dispersed into fine droplets by oxygen (O_2_ 5.0, dispersion gas) supplied at a volume flow of 3.0 L min^–1^ through a surrounding annular gap at a constant pressure drop (Swagelok PTI-E pressure transducer) of 2.0 bar at the nozzle tip. The fuel-precursor mist is ignited by a concentrically arranged CH_4_/O_2_ (CH_4_ 4.5, 1.5 L min^–1^, O_2_ 5.0, 2.0 L min^–1^) combustion flame (supporting flame) surrounding the nozzle exit and converted into oxide monomers and volatile byproducts. Oxygen-rich environments for the synthesis of stoichiometric oxides and to avoid secondary byproducts are provided by an oxygen sheath flow (sheath gas, O_2_ 5.0, 5.0 L min^–1^) guided through a sintered metal plate ring. Constant gas flow rates are ensured via calibrated mass flow controllers (Bronkhorst EL-FLOW). A vacuum pump (Busch Seco SV 1040 C) ensures particle flow downstream the reactor toward the particle collection unit that consists of a glass fiber filter located on a water-cooled filter holder (Hahnemühle, GF6, Ø 257 mm). The exhaust gas temperature is kept below 353 K and controlled with a water-cooled heat exchanger.

### 2.2 Preparation of FSP-Precursor Solutions

For precursor preparation of undoped MgO, the Mg-concentration was set to 0.75 mol L^–1^ by dissolving 16.08 g of magnesium acetate tetrahydrate [Mg(C_2_H_3_O_2_)_2_·4H_2_O, 99%, Strem Chemicals] in 33.4 mL of 2-ethylhexanoic acid (2-EHA, > 99%, Sigma-Aldrich/Merck) and 20 mL of methanol (≥99.8%, Sigma-Aldrich/Merck) under vigorous stirring and filled with a certain amount of xylene (≥98.5%, VWR Chemicals) within a 100 mL batch.

For the synthesis of Ca_*x*_Mg_1–*x*_O and Ba_*x*_Mg_1–*x*_O nanoparticles, the metal concentration within the Ca- and Ba-precursor solution was set to 0.34 mol L^–1^ by dissolving either calcium acetate monohydrate [Ca(C_2_H_3_O_2_)_2_·H_2_O, 98%, Strem Chemicals] at room temperature or barium acetate [Ba(C_2_H_3_O_2_)_2_, 99%, Strem Chemicals] at 120 °C for 5 h using a reflux condenser, under vigorous stirring in 2-EHA. Respective volume ratios of Ca- or Ba-solutions were mixed to the Mg-precursor solution to achieve the desired concentrations of admixtures.

### 2.3 Powder Compaction and Sintering

Compaction of FSP-grown nanocrystals was performed by transferring a defined mass of the powder (*m* = 150 ± 10 mg) into the cavity (*d* = 13 mm) of a compaction tool (FTIR Pellet Dies, Specac) followed by uniaxial compression (*p* = 74 MPa, *t* = 1 min) with a hydraulic press (Atlas Manual Hydraulic Press 15T, Specac). Disk-shaped green bodies are reproducibly obtained in this way, whereby porosities *Φ* of green and sintered compacts can be calculated geometrically through the weight and volume of the pellets via eq 1
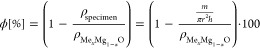
1

To account for the volume fraction of admixed metal ions in the theoretical density values of Me_*x*_Mg_1–*x*_O systems, we applied the *rule of mixture* (see the Supporting Information).

Pressureless sintering of the green bodies was performed within a horizontally operated high-temperature ceramic tube furnace (Nabertherm RHTH80-300/16). Disk-shaped green bodies were sandwiched between alumina plates to guarantee smooth ceramic surfaces after sintering and placed on an alumina crucible in the middle of the tube furnace. Synthesis-related carbonaceous species become eliminated via application of a continuous flow of molecular oxygen [*Q*(O_2_) = 50 mL min^–1^] during the sintering protocol. Specimens were heated with 5 K min^–1^ to the final temperature of 1373 K, dwelled at this temperature for 2.5 h, and then the furnace cooled to room temperature.

### 2.4 X-Ray Diffraction

Continuous scan X-ray diffraction (XRD) data were collected at room temperature in coupled theta–theta mode on a Bruker D8 ADVANCE with DaVinci design diffractometer having a goniometer radius of 280 mm and equipped with a fast-solid-state Lynxeye detector and an automatic sample changer. The specimens were dry-ground in a porcelain mortar and prepared on a single-crystal silicon zero-background sample holder. Data acquisition was done using Cu K_α1,2_ radiation (λ = 154 pm) between 5 and 80.5° *2Θ* with a step size of 0.02° and opened divergence and anti-scatter slits at 0.3 and 4°, respectively. A primary and secondary side 2.5° Soller slit was used to minimize axial divergence and a detector window opening angle of 2.93° was chosen. Data handling and qualitative phase analysis was performed with the Bruker software DIFFRAC.EVA V5.0. The Rietveld program TOPAS 4.2 (Bruker 2012) was used to determine the crystallite domain sizes *d*(hkl) by modeling the intrinsic peak shape of the Bragg peaks by applying the fundamental parameter approach.

### 2.5 Transmission Electron Microscopy

Transmission electron microscopy (TEM) data were acquired using a JEOL JEM-F200 transmission electron microscope operating at 200 kV equipped with a cold field emission electron source and a large windowless JEOL Centurio EDX (energy-dispersive X-ray emission) detector (100 mm^2^, 0.97 srad, energy resolution < 133 eV @ Mn Kα) for local composition analysis. Scanning TEM (STEM) images, such as secondary and back scattered electron or high-angle annular dark-field (HAADF) images showing z-contrast as well as EDX intensity maps were acquired in STEM mode with a typical beam current of 0.1 nA and a beam diameter of 0.16 nm during an acquisition time between 10 and 30 min. The maps were obtained by signal integration of counts over the Mg Kα transition line for Mg (integration: 1.16–1.34 keV), the Ca Kα line for Ca (integration: 3.56–3.82 keV), and the Ba Lα line for Ba (integration: 4.33–4.60 keV). TEM as well as HR-TEM and selected area electron diffraction (SAED) images to access morphological and structural information were recorded using a TVIPS F216 2k by 2k CMOS camera. TEM grids for investigations on particulate samples (see the Supporting Information) were prepared by dipping a lacey carbon grid into the powder in order to investigate structural features and composition of the material adhering to the grid. Evaluation of images acquired during TEM analysis was performed with either ImageJ (V1.52a) or the EM Measure software from TVIPS.

### 2.6 TEM Specimen Preparation

For microstructural TEM analysis on doped MgO ceramics, the specimen was prepared in a three-step approach. The bulk ceramic sample was cut with a razor blade to a final size of approximately 2 by 2 mm with a thickness of around 700 μm. In the second step, thinning of the specimen down to a thickness of around 20 μm was performed with a precision polishing system (Allied MultiPrepTM). Lubrication-free grinding was conducted with diamond-coated polymer films with a roughness ranging from 15 μm down to 0.1 μm at rotating speeds of 10–50 rpm. In the last step, the specimen was polished with argon ions inside a precision ion polishing system (PIPS II, Gatan) operating at a background pressure of *p*(Ar) = 4 · 10^–5^ mbar and constant sample cooling down to 273 K. An energy of 6 keV with a glancing angle of ±5° was applied until the formation of a hole in the specimen’s center occured. The thicknessof the specimen around this hole is below 100 nm, which is suitable for high-quality TEM investigations. To remove artifacts in the specimen arising from the high-energy argon ions, the damaged regions were removed by a stepwise reduction of the argon beam’s energy (3, 1.5, 0.75, 0.37 and 0.1 keV). The last two steps were conducted at a glancing angle of ±10°.

### 2.7 Scanning Electron Microscopy

Imaging of ceramic fracture surfaces was performed with a scanning electron microscope (Zeiss Fe-Ultra Plus 55) equipped with a field-emission gun, Gemini lenses, and an Oxford Instruments EDX-Silicon drift detector (50 mm^2^, energy resolution < 127 eV @ Mn Kα). Secondary electron (SE) images providing topological information and backscattered electron (BSE) images showing z-contrast (see the Supporting Information) were acquired at short working distances of around 3 mm and an accelerating voltage between 5 and 20 kV, with an InLens (ILs) SE and AsB (Angular selective Backscatter) detector, respectively. To limit charging, the scanning electron microscopy (SEM) samples were coated with a few nanometer-thick layer of carbon with a Cressington Carbon Coater 108 carbon/A (2 × 15 s, 4.0 V, background pressure 0.06 mbar) and additionally adhered to the sample holder with copper tape. Image as well as grain size analysis was performed with ImageJ (V1.52a) and the SmartTiff (V3.0) software package from Zeiss.

### 2.8 Photoluminescence Spectroscopy

PL emission spectra at room temperature were recorded on an Edinburgh Instruments FLS 980 PL spectrometer equipped with a double grating monochromator system on both excitation and emission arms. A continuous wave 450 W ozone-free Xe-arc lamp was used as an excitation light source. The front-facing sample holder measuring assembly was chosen to detect emission from the same side of illumination with an R928P PMT detector from Hamamatsu operating at 253 K for optimal dark count reduction. Excitation wavelengths of 270 and 340 nm were chosen to access AEO-specific emission features^30^ by using a slit width of 1 nm for both excitation and emission slits to record the emission signal in steps of 2 nm up to 800 nm. High-vacuum tight-fused silica cells equipped with optical cuvettes allow for a final re-annealing step of ceramic fragments up to 1173 K under defined gas atmospheres [*p*(O_2_) < 10^–5^ mbar, *p*(O_2_) = 650 mbar] to purify and dehydroxylate the external surfaces without breaking the vacuum prior to optical measurements. In order to investigate surface excitonic luminescence, the ceramic specimens were measured under both static high vacuum conditions [*p*(O_2_) < 10^–5^ mbar] and pure oxygen [*p*(O_2_) = 100 mbar] as a PL quencher.

## 3 Results and Discussion

### 3.1 Structure and Microstructure

We used FSP for the synthesis of the metal oxide nanoparticle powders.^40^ These high-surface-area powders with specific surface areas in the range between 230 and 350 m^2^ g^–1^ (Supporting Information, Table S1, ref (40)) were converted into compacts by uniaxial pressing and sintering. With grain sizes below 12 nm (Figure S1, Supporting Information) the particles do also exhibit a uniform distribution of the respective admixtures over the entire ensemble.^40−42^ The advantage of small average grain size, narrow particle size distribution, and uniform distribution of admixtures is - compared to micro- and single-crystalline materials - related to the significantly smaller diffusion paths inside the nanocrystals the admixed ions have to overcome to reach the nanocrystal surface. Ultimately, this should also affect the consecutive reactive behavior of the admixtures inside the intergranular region. Thus, the variety of resulting microstructural features related to doping of the interface region should be more homogeneously distributed over a given piece of ceramic than for ceramics where coarse-grained particle powders have been employed as starting materials.

**Figure 1:**
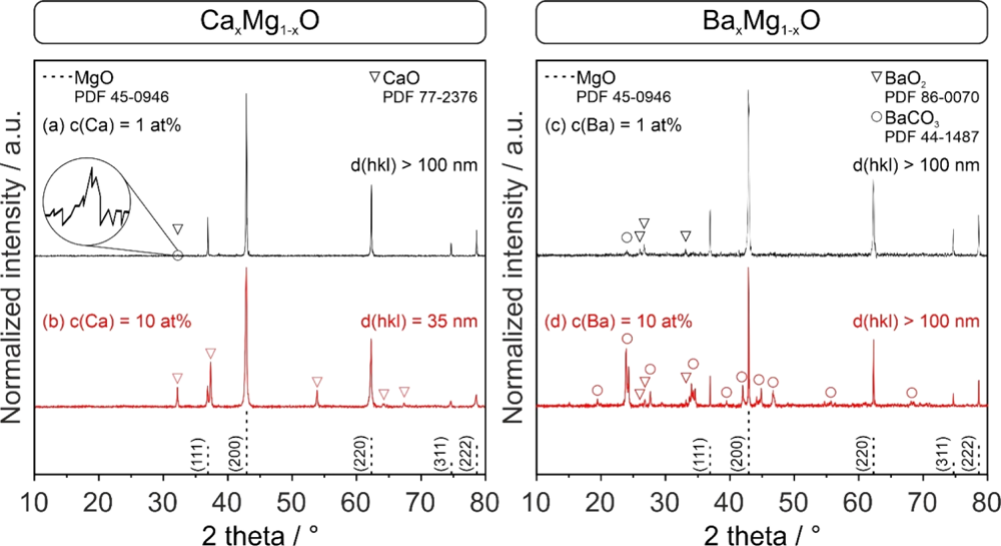
X-ray diffraction patterns of Ca_*x*_Mg_1–*x*_O (left) and Ba_*x*_Mg_1–*x*_O (right) ceramics with admixture concentrations of 1 at. % (a, c) and 10 at. % (b, d). The inset in (a) magnifies a low intense diffraction feature that—on the basis of a complementary TEM-ED analysis (Figure 2)—is attributed to crystalline CaO.

Figure 1 shows XRD patterns of sintered MgO-based compacts with Ca^2+^- and Ba^2+^-admixtures (1 and 10 at. %) after treatment at 1373 K.

**Figure 2:**
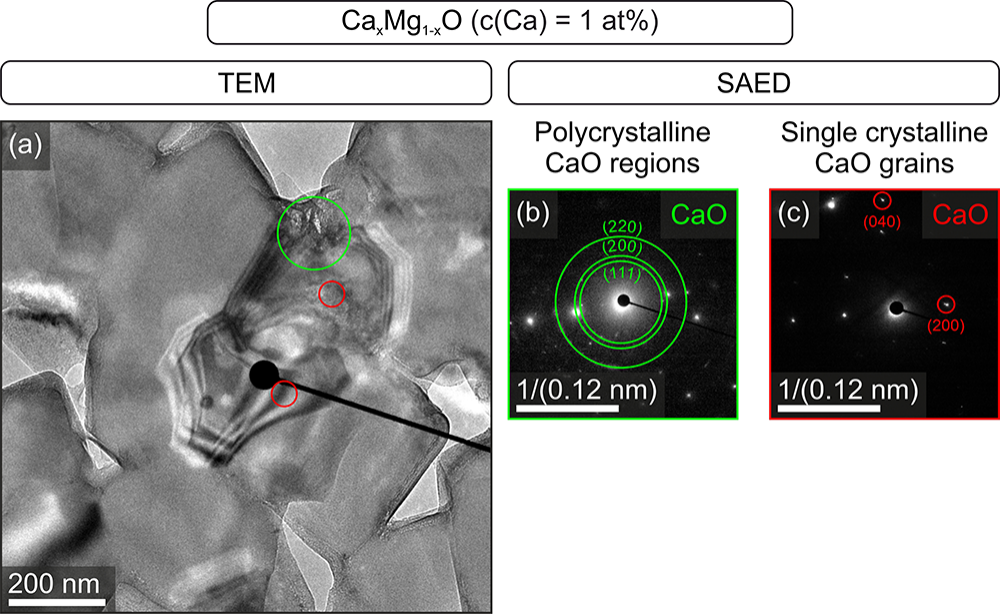
Evidence for the formation of a CaO phase, which results from segregation of Ca^2+^ ions at the interface. Bright field transmission electron micrograph of a Ca_*x*_Mg_1–*x*_O [*c*(Ca) = 1 at. %] ceramic sintered at 1373 K (a, left) with SAED images (b, c right). The green and red circles in (a) indicate regions containing polycrystalline (b, green circle) and single crystalline (c, red circles) CaO inside the intergranular region of the MgO-based sintered compacts.

Apart from nanocrystalline Ca_*x*_Mg_1–*x*_O samples with Ca^2+^-concentrations of 10 at. % (Figure 1b) with an average crystallite domain size of *d*(hkl) = 35 nm, the diffraction patterns of all other Ca^2+^- and Ba^2+^-doped structures contain intense sharp diffraction features, which reflects that the average crystallite domain sizes are above 100 nm. In addition to the prevailing cubic rock-salt structure of the MgO host, the Ca^2+^ ion admixture results in the emergence of additional CaO-specific diffraction features of lower intensity (indicated by ∇ in the left panel of Figure 1). A very weak diffraction feature at *2Θ* = 32.2° can even be traced in samples with Ca^2+^-concentrations as low as 1 at. %. They are attributed to nanocrystalline CaO inclusions that emerge from segregation and subsequent phase separation at the interface. In fact, the presence of minor traces of a nanocrystalline CaO phase could have been confirmed by complementary SAED measurements (Figure 2) taken at different intergranular positions (Figure 2b,c).

The XRD patterns related to the Ba^2+^-doped MgO samples indicate the presence of multiple crystallographic phases.^43,44^ The evidence for barium peroxide and carbonate reflects the increased surface basicity of Ba^2+^-segregates.^44−47^

**Figure 3:**
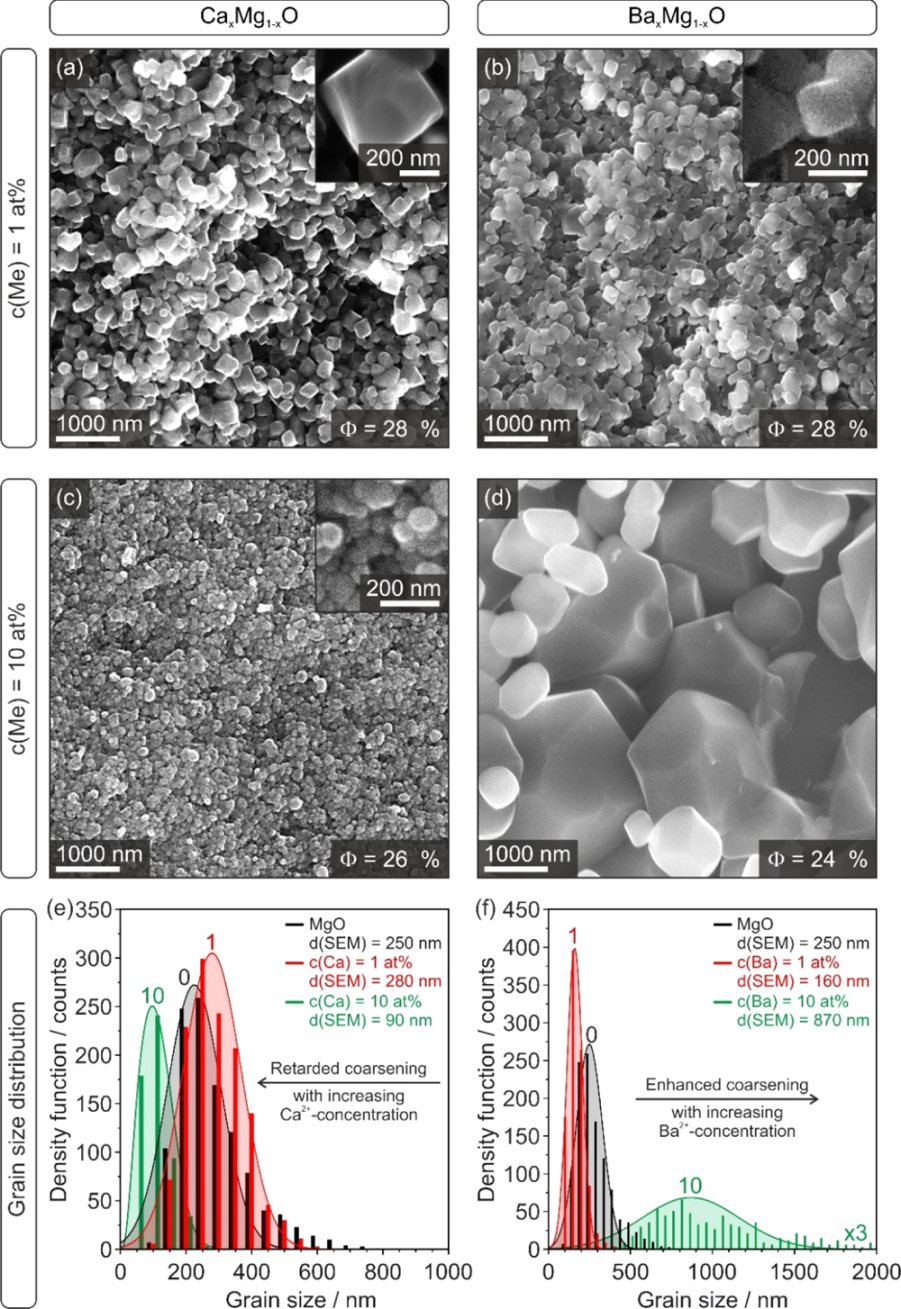
SEM micrographs of Ca_*x*_Mg_1–*x*_O (left column) and Ba_*x*_Mg_1–*x*_O (right column) ceramic fracture surfaces. The top rows show images from surface topology analysis with the SE ILs detector. Information about residual porosity (*Φ*) is added to the right corner at the bottom of the micrographs (a–d). Grain size distribution plots in (e, f) do also include those of MgO without the admixture as a reference (Supporting Information, Figure S4).

SEM measurements on Ca^2+^- and Ba^2+^-doped ceramic fracture surfaces (Figure 3) were performed to assess grain sizes and morphologies and to compare these observations with ceramics derived from MgO nanocube powders without admixtures.

Residual porosities *Φ* in the range of 24–28% are consistent with the relatively open microstructure that can be evidenced by the SEM images (Figure 3). Very different trends in the evolution of grain size and morphology were observed for the Ca_*x*_Mg_1–*x*_O and Ba_*x*_Mg_1–*x*_O ceramics, that is, on materials that were derived from comparable nanoparticle powders with equal particle size distributions (Supporting Information, Figures S1–S3) and the same high level of homogeneously distributed admixed ions.

Grain morphology and size distribution of samples with the Ca^2+^-admixture are homogeneously distributed. Whereas a Ca^2+^-concentration of 1 at. % (Figure 3a) predominantly exhibit grains with a cubic habit and soft edges, samples with a Ca^2+^-concentration of 10 at. % (Figure 3c) are made up of significantly smaller grains (Figure 3e) of less defined morphologies. Comparable to MgO with a median grain size of *d*(SEM) = 250 nm, the Ca^2+^-admixture of 1 at. % leads to a grain size distribution with a median size [*d*(SEM) = 280 nm]. After sintering at 1373 K, samples with Ca^2+^-concentrations of 10 at. %, however, must have experienced suppressed grain growth and coarsening, leading to a remarkably smaller median grain size of *d*(SEM) = 90 nm, that is, a value that is significantly below that of grains of MgO ceramics.

The trend for the Ba_*x*_Mg_1–*x*_O ceramic is distinctly different: the grains show a cubic habit, exhibit soft edges, and are homogeneously distributed for a Ba^2+^-concentration of 1 at. % (Figure 3b). They are also significantly smaller in size as compared to ceramics with 1 at. % Ca^2+^. An increased Ba^2+^-loading of 10 at. % (Figure 3d) produces more inhomogeneous sample properties such as mixtures of small grains with rounded edges, on the one hand, and larger polyhedral grains, on the other. The grain sizes for a *c*(Ba) = 1 at. % sample are characterized by a narrow distribution function, whereas the one for a *c*(Ba) = 10 at. % sample is broad with grains reaching sizes of 2 μm and with corresponding median values of *d*(SEM) = 160 nm and *d*(SEM) = 870 nm, respectively. Thus, the trend of sintering induced grain growth is very different for Ca_*x*_Mg_1–*x*_O and Ba_*x*_Mg_1–*x*_O ceramics that are derived from comparable nanoparticle powders with *d*(TEM) < 12 nm (Supporting Information, Figures S1–S3 and Table S1).

### 3.2 Intergranular Distribution of CaO and BaO Segregates

**Figure 4:**
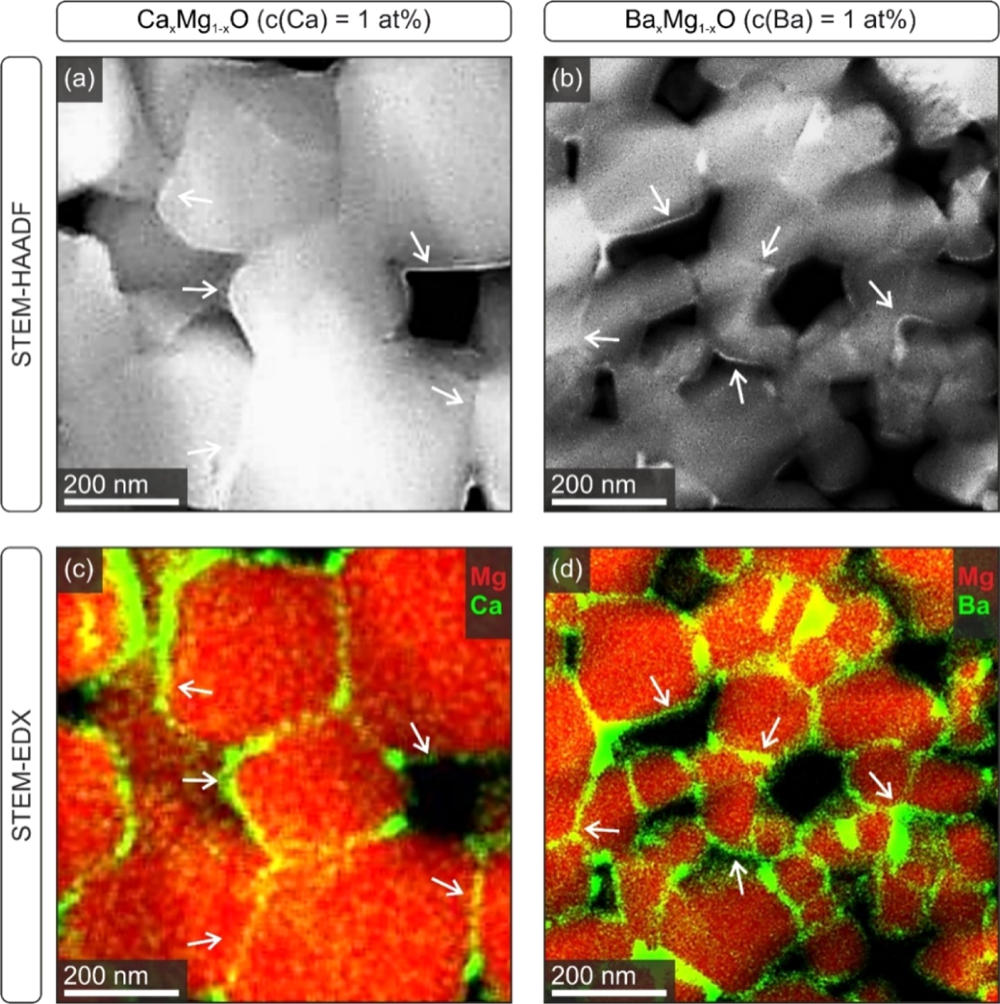
TEM analysis of Ca- and Ba-segregation in Ca_*x*_Mg_1–*x*_O [*c*(Ca) = 1 at. %, left a, c] and Ba_*x*_Mg_1–*x*_O [*c*(Ba) = 1 at. %, right b, d] compacts after sintering at 1373 K. STEM–HAADF images (first row a, b) provide information about the compositional contrast. The white arrows indicate regions of Ca- and Ba-accumulation as supported by STEM–EDX intensity maps (second row c, d).

A detailed TEM analysis using STEM and EDX mapping revealed the dispersion of CaO and BaO in the ceramic microstructures after powder compaction and sintering. Figure 4 shows STEM–HAADF images (first row) and corresponding EDX intensity maps (second row) recorded on Ca_*x*_Mg_1–*x*_O (left) and Ba_*x*_Mg_1–*x*_O (right) compacts with admixture concentrations of 1 at. %.

In addition to contrasts in brightness that originate from sample thickness in the HAADF images (Figure 4a, b), there also exist bright regions (indicated by arrows) that originate from the higher compositional contrast of the Ca^2+^- and Ba^2+^-segregates. These segregates deposit both at MgO-based grain surfaces and their intergranular interfaces. From the HAADF images shown here, we determined values for film thicknesses of 9 ± 1 and 5 ± 1 nm for samples with *c*(Ca) = 1 at. % or *c*(Ba) = 1 at. %, respectively. Such values were found to be consistent with those determined for the segregate films in other sample regions, though - due to the lack of a sufficient number of sample locations - statistically not significant. EDX intensity maps (second row) clearly prove the presence of thin Ca^2+^- and Ba^2+^-rich segregate regions at free pore surfaces and within GB phases.

### 3.3 Discussion of the Grain Size Evolution Trends Observed

The most striking result corresponds to the very different stabilities (Figure 3) of the doped MgO grains following the order:
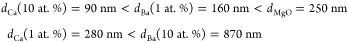


Whereas the median value for the grain size distribution for Ca_*x*_Mg_1–*x*_O compacts decreases with higher Ca^2+^-concentrations with *c*(Ca) = 10 at. %, it increases for Ba_*x*_Mg_1–*x*_O compacts with *c*(Ba) = 10 at. %. To understand the impact of the Ca^2+^- or Ba^2+^-admixture on grain growth and to rationalize the opposite trends observed, one needs to consider different material- and process-specific factors that can come into play.

In crystalline systems, four types of driving forces have been recognized as reasons for interface segregation: these are, the difference in surface energy between the admixed oxide and the host, an elastic solute strain energy due to the size difference between the admixed ions and host ions, solute–solvent interaction, and electrostatic potential/charge compensation.^48^ For the present materials’ situation, we suggest that the following three factors contribute predominantly to admixture segregation and consequently determine associated grain growth at elevated temperatures.

#### 3.3.1 Surface and Interface Energy Changes

Both theoretical and experimental work revealed that the surface segregation of Ca^2+^- and Ba^2+^-ions into the MgO surface is associated with surface energy reductions (Table 1). Tasker et al.^12^ showed that Ca^2+^, Sr^2+^, and Ba^2+^ will preferentially segregate to the MgO (100) surfaces. Admixed ions will segregate in the interfaces to decrease the interface energies and, thus, the overall energy of the system. Lower values for doped specific surface or GB energies are associated with the interface enrichment of admixed ions, which reflects one important driving force for particle coarsening.

**Table 1:** Segregation Energies and Dopant-Induced Surface Energy Change δγ of (001) Surfaces on MgO (γ_001_ = 1.06 J m^–2^), as a Function of Ca^2+^, Sr^2+^, and Ba^2+^ Coverage^12^

	segregation energy/kJ·mol^–1^			surface energy change δγ/J·m^–2^		
coverage	Ca^2+^	Sr^2+^	Ba^2+^	Ca^2+^	Sr^2+^	Ba^2+^
isolated impurity (infinite dilution)	–104.19	–215.14	–376.25			
1/4 coverage	–81.04	–162.08	–288.46	–0.36	–0.71	–1.27
1/2 coverage	–46.31	–62.71	–107.09	–0.41	–0.54	–0.95
full coverage	–38.59	–25.08	+28.94			
reconstructed monolayer	–46.31	–108.05	–136.03	–0.82	–1.90	–2.40^a^

a Note that BaO grown on MgO(100) forms three-dimensional islands, as monoatomic BaO layers on MgO are unstable in comparison to BaO bilayers.^43,49^ Moreover, the potential emergence of new types of solid–solid interfaces was not taken into account within the calculations.

Rohrer and co-workers,^50,51^ studied the segregation behavior of Ca^2+^- and Ba^2+^-solutes in polycrystalline MgO and concluded from their experimental work that segregation lowers GB energies. The level of GB segregation was found to be anisotropic. Thus, the admixture of interfacial active impurities should alter the relative abundance with which GBs of different types appear. For MgO samples with Ca^2+^- and Ba^2+^-admixtures, however, Rohrer et al.^50^ found that none of the impurities has an effect on the grain orientation or misorientation texture. In other words, over the whole domain of crystal orientations, the populations of grains were equally distributed. In fact, Ca^2+^ segregation at GBs has a much stronger impact on changing the GB plane distribution in MgO compacts than dopants such as Sr^2+^, Ba^2+^, or Y^3+^, which - due to their size and/or charge mismatch - must segregate more strongly into the GBs than Ca^2+^. Thus, specifically Ca^2+^-segregation in MgO decreases the energy of the lowest energy {100} GBs. This has been attributed to the smaller radius of the Ca^2+^-ions as compared to Sr^2+^- and Ba^2+^-ions, which become more easily accommodated at GBs with at least one {100} terminating plane than at other GBs. Ba^2+^-ions, on the other hand, exhibit much higher misfit energies at all GBs and are more evenly distributed over GBs of all orientations.

Considering the impurity-induced changes in the surface energies (Table 1) and GB energies, one can expect for thermodynamic equilibrium conditions that the grain size stability increases in the order of CaO(surface)/MgO > BaO(surface)/MgO > MgO, which would explain the significantly smaller grain sizes for the 10 at. % Ca^2+^ and 1 at. % Ba^2+^ samples. It, however, does not explain the opposite trends in grain size evolution with impurity concentration.

As a result of the conditions prevailing during the gas phase synthesis where the Ca–O and Ba–O moieties become randomly trapped inside the host lattice prior to temperature quenching - within a few milliseconds - from 2000 K to room temperature, the here described materials’ situation is far from thermodynamic equilibrium. The concentrations of admixtures are significantly higher and beyond the solubility limit below 1 at. % for Ba^2+^ and approximately corresponds to 1 at. % for Ca^2+^ (Figure S5 in the Supporting Information).^52,53^ In addition, ion vacancies substantially impact ion diffusion and associated crystallite reorganization (see below). As a result of the non-equilibrium synthesis approach employed here, the actual defect concentrations must be significantly higher than those in extended crystals in thermodynamic equilibrium.

Subsequent vacuum annealing to 873 or 1173 K enables the partial reorganization of size, shape, and compositional distribution of the individual crystallites. Impurity diffusion and segregation give rise to concentration gradients of Ba^2+^- or Ca^2+^-ions with their depletion in the bulk and enrichment at the grain surfaces and interfaces.

#### 3.3.2 Ion Diffusion in MgO Crystals: Diffusion Coefficients and Diffusion Barriers

Van Orman and Crispin comprehensively reviewed data on ion diffusion in MgO crystals.^54^ Apart from Be^2+^, which follows an interstitial diffusion mechanism, all remaining divalent alkaline earth-metal cations move through the MgO lattice via an ion vacancy mechanism. Consequently, the experimentally determined diffusion data do sensitively depend on the concentration of cation vacancies, which can be intrinsic or extrinsic, that is, stabilized by trivalent impurity cations.^55^ As outlined above, the AEO nanoparticles were grown under non-equilibrium conditions,^40^ leading to defect concentrations that are above corresponding equilibrium values. Thus, the diffusion coefficients reported for cation diffusion in single crystals with cation vacancy concentrations of up to 500 ppm (*D*_Mg_ = 10^–19^ m^2^ s^–1^, *D*_Ba_ = 10^–21^ m^2^ s^–1^ and *D*_Ca_ = (10^–18^–10^–19^) m^2^ s^–1^ at temperatures around *T* = 1173 K)^54^ do not apply for the nanocrystals that are supersaturated in Ca^2+^- and Ba^2+^-ions as well as in ion vacancies.

A more appropriate measure for such a thermodynamically metastable situation is the computed values for the diffusion barriers of Mg^2+^ and impurity ions in the MgO host lattice.^43^ In fact, the barrier for Ba^2+^-diffusion was found to be significantly decreased because larger Ba^2+^-ions are incorporated in highly unstable local environments. Thus, a considerable part of the O–O expansion that is required in the diffusion event is already realized in the initial configuration.^43^ Whereas the barrier for Ba^2+^-diffusion in the MgO lattice was calculated to be 0.54 eV, the respective value for a Mg^2+^-ion is by a factor of 4 higher. The barriers for the diffusion of Ca^2+^ and Sr^2+^ were computed to be 2.2 and 1.63 eV, respectively. The more pronounced size mismatch between the Ba^2+^-ions and the Mg^2+^-ions together with the lower barrier for Ba^2+^-migration inside the MgO host lattice suggest at least for the 1 at. % samples that sintering drives more effectively Ba^2+^-ions than Ca^2+^-ions into the surface of the MgO grains.

#### 3.3.3 Admixture Loading and the Difference of Ca^2+^- and Ba^2+^-solubility

**Figure 5:**
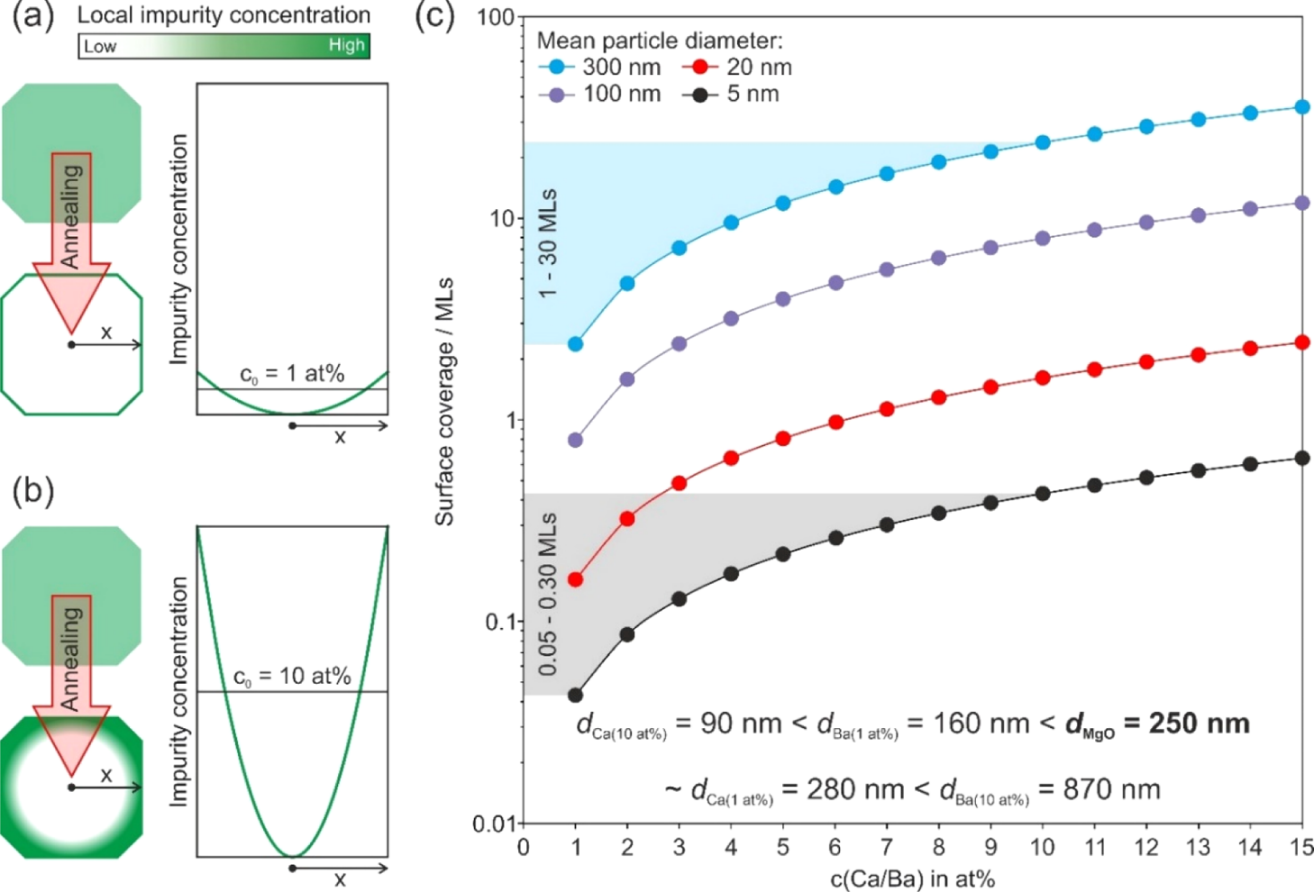
Initial impurity ion concentration and surface coverage on MgO grains after complete segregation. Schemes (a, b) illustrate the annealing-induced surface diffusion for initial impurity concentrations of 1 at. % (a) and 10 at. % (b), strongly enhanced for higher initial admixture concentrations. Note that the profiles in (a, b) cannot account for the residual impurity ion solubility in the MgO lattice, being larger for Ca^2+^-ions than for Ba^2+^-ions. The estimated mono- and multilayer coverages for grains with initial diameters of 5, 20, 100, and 300 nm as a function of impurity loading are plotted in (c). Details of the underlying calculations in (c) are provided in the Supporting Information.

Upon annealing-induced segregation of impurity ions, their spatial distribution inside the grains changes. Related concentration profiles (Figure 5a,b) convert from flat lines representing equal concentrations *c*_0_ throughout the particle, which characterizes the random distribution of admixtures right after particle quenching, to parabolic ones after segregation.

As a result of size mismatch and associated local strain effects inside the bulk, Ca^2+^- and Ba^2+^-ions segregate into the surface where they - depending on their wetting properties - form surface structures between uniform layers and metal oxide clusters. Grain coarsening occurs in parallel to segregation and a rough estimate of how the segregate’s surface coverage depends on grain size (Figure 5c) illustrates that - under the ideal assumptions of (i) complete impurity segregation and (ii) perfect wetting of the segregated CaO or BaO layers - the number of uniform layers range from a submonolayer coverage (for nanocrystals) to several tens of a monolayer (for grains in the size regime between 200 and 300 nm). The emergence of segregate multilayers implies that interface energies related to the contact areas between MgO and the CaO or BaO intergranular films need to be included in future studies. Note that the size of these intergranular films, as determined from the analysis of the HAADF images (Figure 4a,b), exceeds the values depicted in Figure 5c by a factor of 10–20. This is not surprising since the real intergranular distribution of impurities is significantly less homogeneous than assumed for the ideal scenario. Some of the grain faces lack segregate-related surface decorations, and others have converted into inclusions filling the intergranular pores. Moreover, previous computations have shown that BaO adlayers on Mg(100) surfaces tend to dewet because the gain by internal mono-oxide cohesion is higher than the cost of interface formation and strongly favors BaO dewetting from the surfaces and interfaces of the MgO-based grains.^43^ In any case, samples with *c*(Ca) = 1 at. % show thicknesses of 5 ± 1 nm, which is about half of that measured for *c*(Ba) = 1 at. % with 9 ± 1 nm, and are consistent with the grain size-dependent trend illustrated by Figure 5c.

Since we have received evidence for CaO precipitate formation, we assume that this has also an impact on the coarsening behavior of the local microstructure. As an alternative and complementary explanation for the inhibited grain growth observed for MgO samples with Ca^2+^ admixtures, small CaO precipitates can block interface motion by the particle pinning effect.^34,48^ The surface chemistry of segregated admixtures has an additional effect on ceramic processing. There is an increase in surface basicity of the grains induced by the Ca^2+^–O^2–^ or Ba^2+^–O^2–^ moieties in the grain surfaces.^45,46^ This, in turn, promotes the adsorption of H_2_O and/or CO_2_ molecules during material compaction and handling in air. BaO moieties at the surface of less reactive grains such as MgO instantaneously react with water to form surface hydroxides.^44^ Additionally adsorbed water forming thin films that cover these highly dispersed structures act as a two-dimensional solvent, facilitates mass transport, and acts as a sintering aid.^56,57^ Thus, the higher the surface basicity and reactivity of the nanocrystalline starting material, the higher is the uptake of water from the ambient. Ba_*x*_Mg_1–*x*_O nanoparticle samples with 10 at. % Ba adsorb more water, which promotes grain growth upon sintering-induced densification.

To some extent, the different trends in grain coarsening do also reflect the overall complexity of sintering, which entails many different mechanistic steps and in the present case also includes segregation and phase separation. (See CaO nanocrystal inclusions entrapped between MgO-based grains in Figure 2.) Even for a manufacturing process that uses extremely homogeneous starting materials (Supporting Information, Figures S1–S3 and Table S1), there are a number of effects that can counteract the homogeneous distribution of final materials properties. These involve temperature gradients inside the sinter zone, formation, closing and opening of pores during annealing, and - consequently - local concentration changes in the environmental gas atmosphere also including the H_2_O partial pressure. Clearly, more measures have to be accounted for to optimize the process and, thus, the final properties of the manufactured ceramics.

### 3.4 PL Light Emission from Grain Surfaces and Interfaces

**Figure 6:**
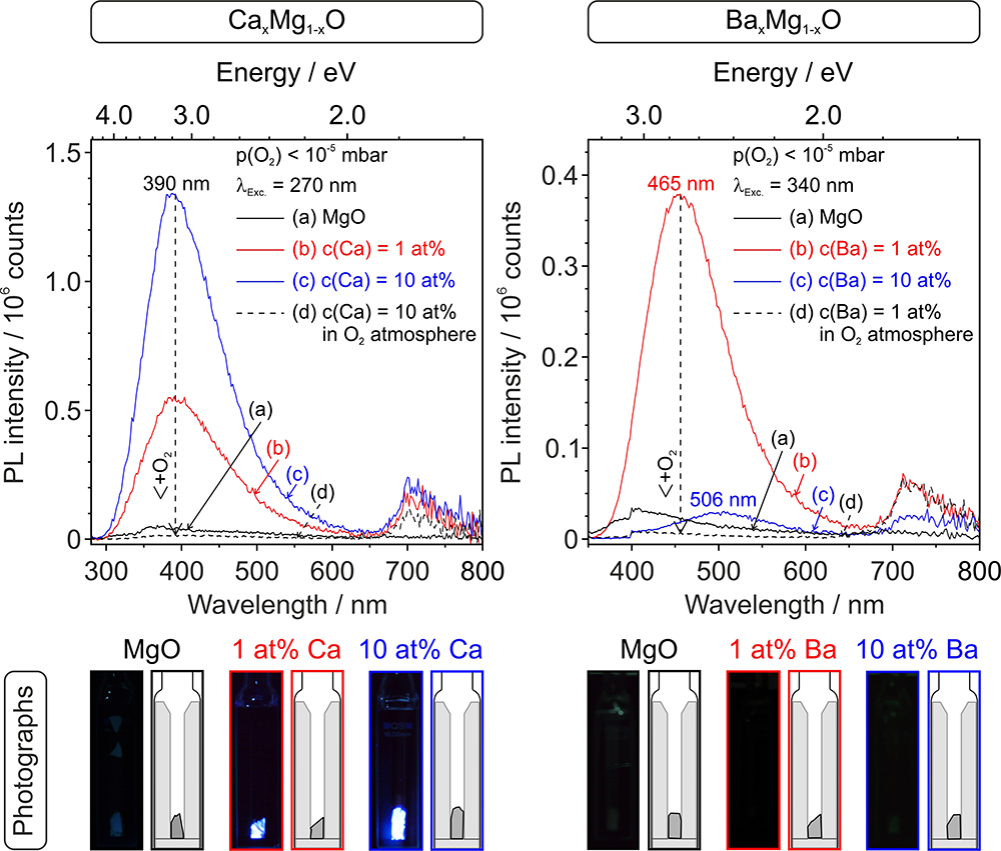
PL emission spectra of 1373 K sintered Ca_*x*_Mg_1–*x*_O (left, λ_Exc._ = 270 nm) and Ba_*x*_Mg_1–*x*_O (right, λ_Exc._ = 340 nm) compacts. Measurements were performed under static high-vacuum conditions (a–c) or in an O_2_ atmosphere [d, *p*(O_2_) = 100 mbar]. The additional emission signal between 700 and 750 nm is attributed to an experimental artifact of so far unresolved origin. The bottom row shows digital images during sample illumination using a UV Xe-lamp in combination with related interference filters.

PL emission spectra were acquired on ceramic fragments using UV-excitation at λ_Exc._ = 270 nm and λ_Exc._ = 340 nm for Ca_*x*_Mg_1–*x*_O (Figure 6, left) and Ba_*x*_Mg_1–*x*_O (Figure 6, right) in vacuum, respectively.

Excitation of MgO ceramics (Figure 6a left and right panel) yields extremely weak emission features. Photoexcitation of Ca^2+^- or Ba^2+^-admixed ceramics, however, produces stronger PL bands of substantially higher intensities. Ca_*x*_Mg_1–*x*_O ceramics exhibit a broad and intense emission feature in the range of 300–600 nm with a maximum at 390 nm. In case of Ba_*x*_Mg_1–*x*_O ceramics, the PL emission bands are red-shifted and in the range between 350 and 650 nm with maxima at 465 and 506 nm for Ba^2+^-concentrations of 1 and 10 at. %, respectively.

Emission intensities of Ca^2+^-admixed ceramics exceed those of Ba_*x*_Mg_1–*x*_O ceramics by roughly one order of magnitude. In contrast to Ca_*x*_Mg_1–*x*_O, where higher Ca^2+^-loadings increase emission intensities (see also digital photographs for Ca_*x*_Mg_1–*x*_O ceramics at the bottom of Figure 6), the opposite effect is observed for a higher Ba^2+^-content [*c*(Ba) = 10 at. %]. Admission of oxygen as a PL quencher annihilates the observed emission bands in all material combinations. This is a reversible effect since subsequent pumping restores the original band intensities. The admixture of both Ca^2+^- and Ba^2+^-ions to MgO leads to PL emission signals that shift from the UV region for MgO to the range of visible light.^28−30,58,59^ Variations in the dispersion of Ca^2+^- and Ba^2+^-ions have an impact on both grain size evolution during sintering and light emission intensities. Enhanced emission properties in samples hosting 1 at. % of Ca^2+^- and Ba^2+^-admixtures result from the higher level of admixture dispersion inside intergranular regions and from smaller grain sizes (Figures 3 and 4). This is reflected by the fact that the Ca^2+^-concentration increase stabilizes the MgO-based grains toward coarsening and limits grain growth to sizes down to *d*(SEM) = 90 nm (Figure 3) with an increased abundance of well-dispersed excitation and emission sites.^30^ The presence of Ba^2+^ in excess promotes coarsening (Figure 3) and formation of larger Ba–O clusters^43^ (see Figure S5) and reduces the concentration of excitation and emission sites. That oxygen admission quenches the photoemission signals clearly proves that the radiative annihilation of excitons occurs preferentially at free grain surfaces. These are accessible to gaseous oxygen, whereas functionalized and inaccessible intergranular regions inside the porous ceramics are not.

## 4 Conclusions

We analyzed the segregation behavior of Ca^2+^- and Ba^2+^-admixtures in MgO nanocrystal-derived compacts after sintering. The nature and concentration of admixtures were varied to control the size and composition of emerging intergranular regions and microstructure evolution. As a result of material densification at 1300 K, where residual porosities between 24 and 28% could have been retained, internal (solid–solid) interfaces and external (solid–gas) surfaces come into existence. HR-TEM in combination with local elemental analysis measurements revealed that Ca^2+^- and Ba^2+^-ions segregate into both types of surfaces and interfaces, where they further impact grain coarsening, densification behavior and - ultimately - the optical properties of the ceramic.

Whereas contact regions between undoped particles in consolidated powders can give rise to new types of defects that can be probed with PL spectroscopy,^60^ this work reveals that only those Ca^2+^- and Ba^2+^-segregates that decorate the pore surfaces and external grain surfaces can actively contribute to PL emission. The stabilization as PL-active inclusions underlines the potential for their implementation in ceramic phosphors. Hence, the densification of vapor phase-grown nanoparticle powders with extremely well-defined bulk and surface properties to generate ceramics can lead to a high abundance of structurally and compositionally uniform intergranular regions that emerge from the interrelated effects of segregation and grain growth.

In addition to their role as excitation and PL emission sites in thermally stable inorganic phosphors with earth-abundant elements, the admixtures of highly dispersed Ca^2+^ and Ba^2+^ also determine grain coarsening and microstructure evolution. Potential limitations which arise from diffusion during segregation can be circumvented. The impact of nature and interface loading with segregates on the driving forces for sintering can be fully exploited for optimization of their functional properties.

## Supporting Information Available

Additional powder characterization data obtained by TEM, XRD, and N_2_-sorption analysis; electron microscopy results (TEM and SEM) showing fracture surface topology of undoped MgO and the elemental distribution of admixtures [*c*(Me) = 10 at. %] in doped MgO ceramics; equilibrium phase diagrams of MgO–CaO and MgO–BaO solid solutions; and details about the performed calculations for approximation of the mixed metal oxide density (rule of mixture) and surface coverage (PDF)
