# Supplementary material for: Segregation Engineering in MgO Nanoparticle-Derived Ceramics: The Impact of Calcium and Barium Admixtures on the Microstructure and Light Emission Properties
Source: ACS Appl Mater Interfaces. 2021 May 19;13(21):25493–502. doi: 10.1021/acsami.1c02931 (PMC8176451; doi:10.1021/acsami.1c02931)
Supplement: Supplementary file 1 — am1c02931_si_001.pdf [file am1c02931_si_001.pdf]

SUPPORTING INFORMATION

Segregation engineering in MgO nanoparticle derived ceramics: The impact of calcium and barium admixture on microstructure and light emission properties

Thomas Schwab, Korbinian Aicher, Hasan Razouq, Gregor A. Zickler, and Oliver Diwald[*]

Department of Chemistry and Physics of Materials
Paris-Lodron University of Salzburg
Jakob-Haringer-Straße 2a
5020 Salzburg, Austria
E-mail: oliver.diwald@sbg.ac.at

- **Table of contents**

**Powder characterization results**	
Figure S1. Transmission electron microscopy data – Particle size distributions	2
Figure S2. Transmission electron microscopy data – Particle morphology of doped MgO nanoparticles	3
Figure S3. Powder X-ray diffraction patterns	4
Table S1. Comparison of particle and crystallite domain sizes obtained from BET, TEM and XRD	5
**Additional electron microscopy results**	
Figure S4. SEM sample topology investigation of an MgO ceramic	6
Figure S5. Elemental distribution of admixtures (*c*(Me) = 10 at%) in doped MgO ceramics	7
**Equilibrium phase diagrams**	
Figure S6. Equilibrium phase diagrams of MgO-CaO and MgO-BaO solid solutions.	8
**Details about performed calculations**	
Rule of mixture	9
Surface coverage	10
**References**	11

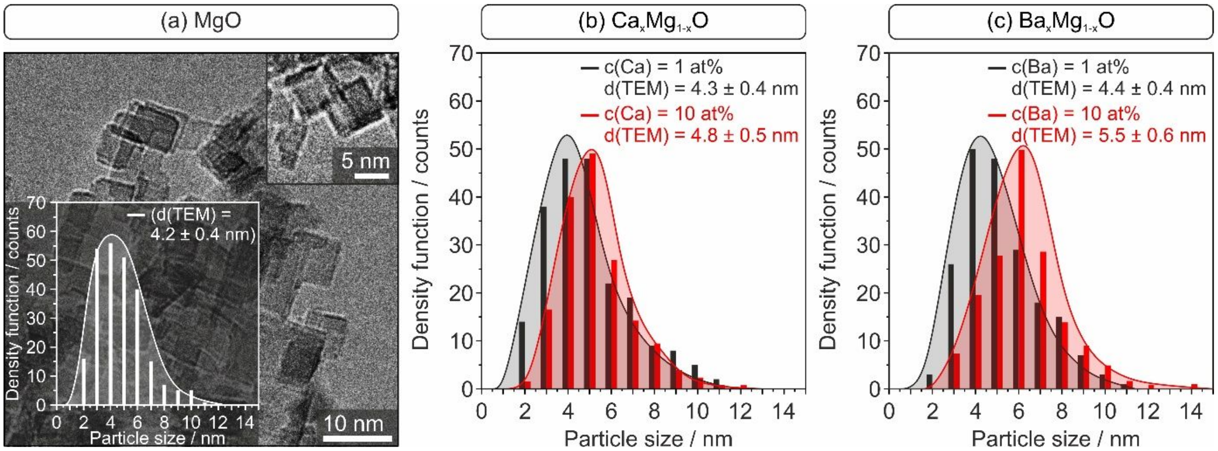

Figure S1. Transmission electron micrograph of as-synthesized MgO nanocubes via FSP (a, left column) with inset of the particle size distribution (PSD) plot and magnified characteristic region of cubic particle habits, compared to PSD plots of $Ca_xMg_{1-x}O$ (b, middle) and $Ba_xMg_{1-x}O$ (c, right). TEM micrographs with insets of characteristic regions of particle morphologies for $Ca_xMg_{1-x}O$ and $Ba_xMg_{1-x}O$ nanoparticles are displayed in Figure S2.

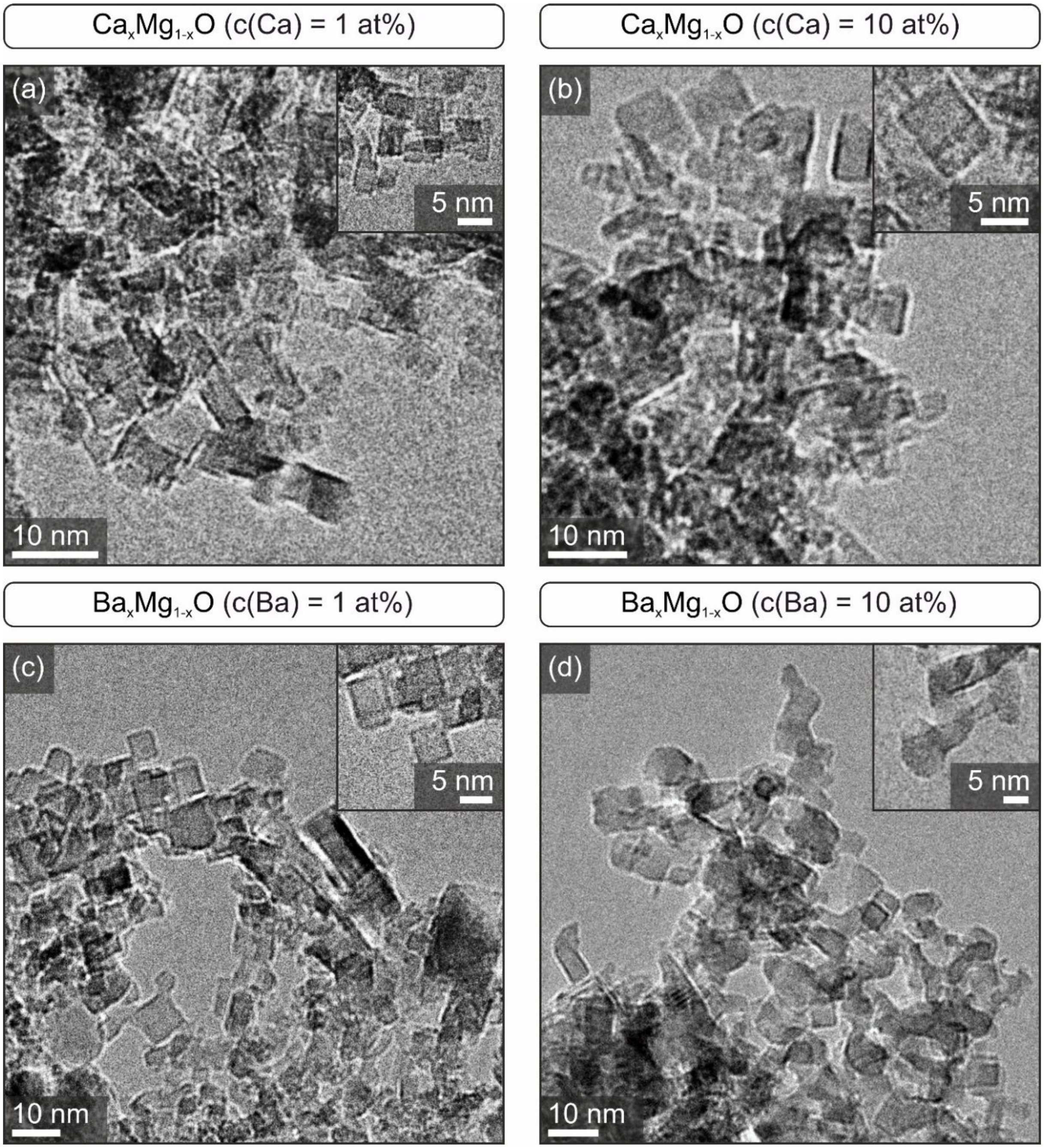

Figure S2. Transmission electron micrographs of as-synthesized $Ca_xMg_{1-x}O$ (first row a, b) and $Ba_xMg_{1-x}O$ (second row c, d) nanoparticles obtained from FSP, with insets of magnified characteristic regions of particle habits for admixture concentrations of 1 at% (a, c) and 10 at% (b, d).

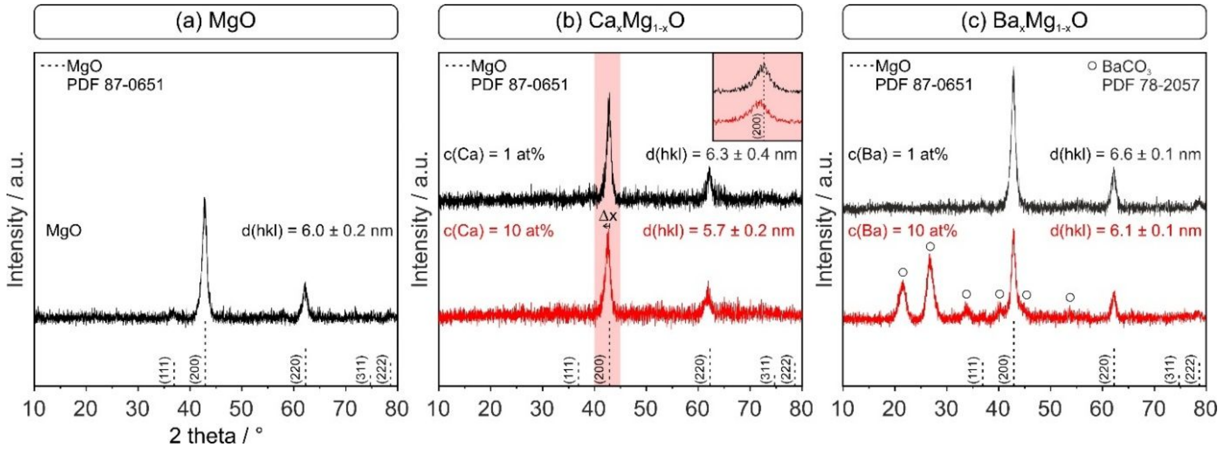

Figure S3. Powder XRD patterns of FSP-synthesized MgO (a, left) compared to those of $Ca_xMg_{1-x}O$ (b, middle) and $Ba_xMg_{1-x}O$ (c, right) nanoparticles with nominal admixture concentrations of 1 at% and 10 at%. The slight shift of reflection features in $Ca_xMg_{1-x}O$ is highlighted and exemplified on the (200) main reflection (b, magnified in the top right inset). Crystallite domain sizes are calculated with the Scherrer equation by evaluation of the MgO related (200) main diffraction feature.

A comparison of particle (*d*(BET), *d*(TEM)) and crystallite sizes (*d*(XRD)) accessed with complementary characterization techniques is summarized in Table S1. Particle sizes calculated from the integral sample volume (*d*(BET)) assuming perfectly shaped and monodisperse cubes and observed in the TEM (*d*(TEM)) analysis are in good consistence and smaller compared to crystallite domain sizes (*d*(XRD)). Thus, clearly proving the abundance of nanopowders composed out of monocrystalline particles.

Table S1. Specific surface area (*S*(BET)) and thereof derived particle sizes obtained from N_2-sorption (*d*(BET)), compared to particle sizes from TEM analysis (*d*(TEM)) and crystallite domain sizes (*d*(XRD)) of undoped MgO and doped $Me_xMg_{1-x}O$ (Me = Ca, Ba) as-synthesized nanocrystalline powder materials with admixture concentrations of 1 at% and 10 at%.

Impurity ion concentration	Oxide system	*S*(BET) / $m^2 \cdot g^{-1}$	*d*(BET) / nm	*d*(TEM) / nm	*d*(XRD) / nm
Undoped	MgO	288 ± 43	5.8 ± 0.9	4.2 ± 0.4	6.0 ± 0.2
c(Me) = 1 at%	$Ca_xMg_{1-x}O$	303 ± 46	5.5 ± 0.8	4.3 ± 0.4	6.3 ± 0.4
	$Ba_xMg_{1-x}O$	264 ± 40	6.3 ± 0.9	4.4 ± 0.4	6.6 ± 0.1
c(Me) = 10 at %	$Ca_xMg_{1-x}O$	230 ± 35	7.4 ± 1.1	4.8 ± 0.5	5.7 ± 0.2
	$Ba_xMg_{1-x}O$	346 ± 52	4.3 ± 0.6	5.5 ± 0.6	6.1 ± 0.1

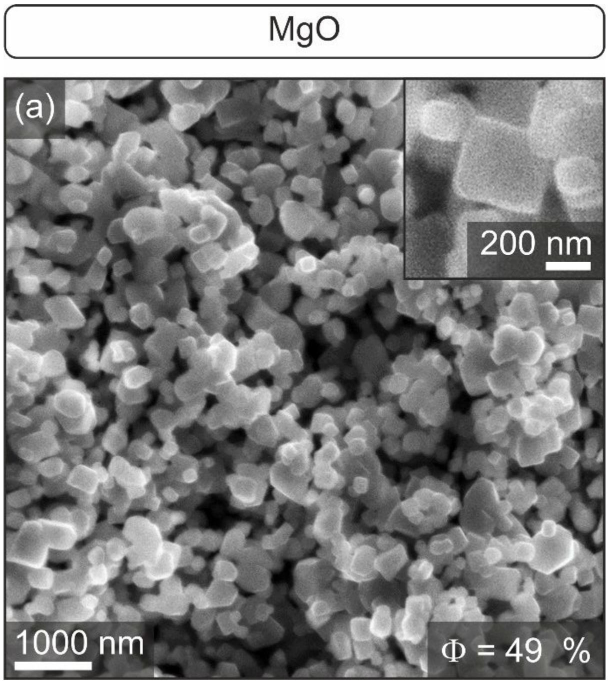

Figure S4. Secondary electron SEM image of an MgO ceramic fracture surface acquired with the IL-detector. Information about residual porosity (Φ) is added to the right corner at the bottom of the micrograph.

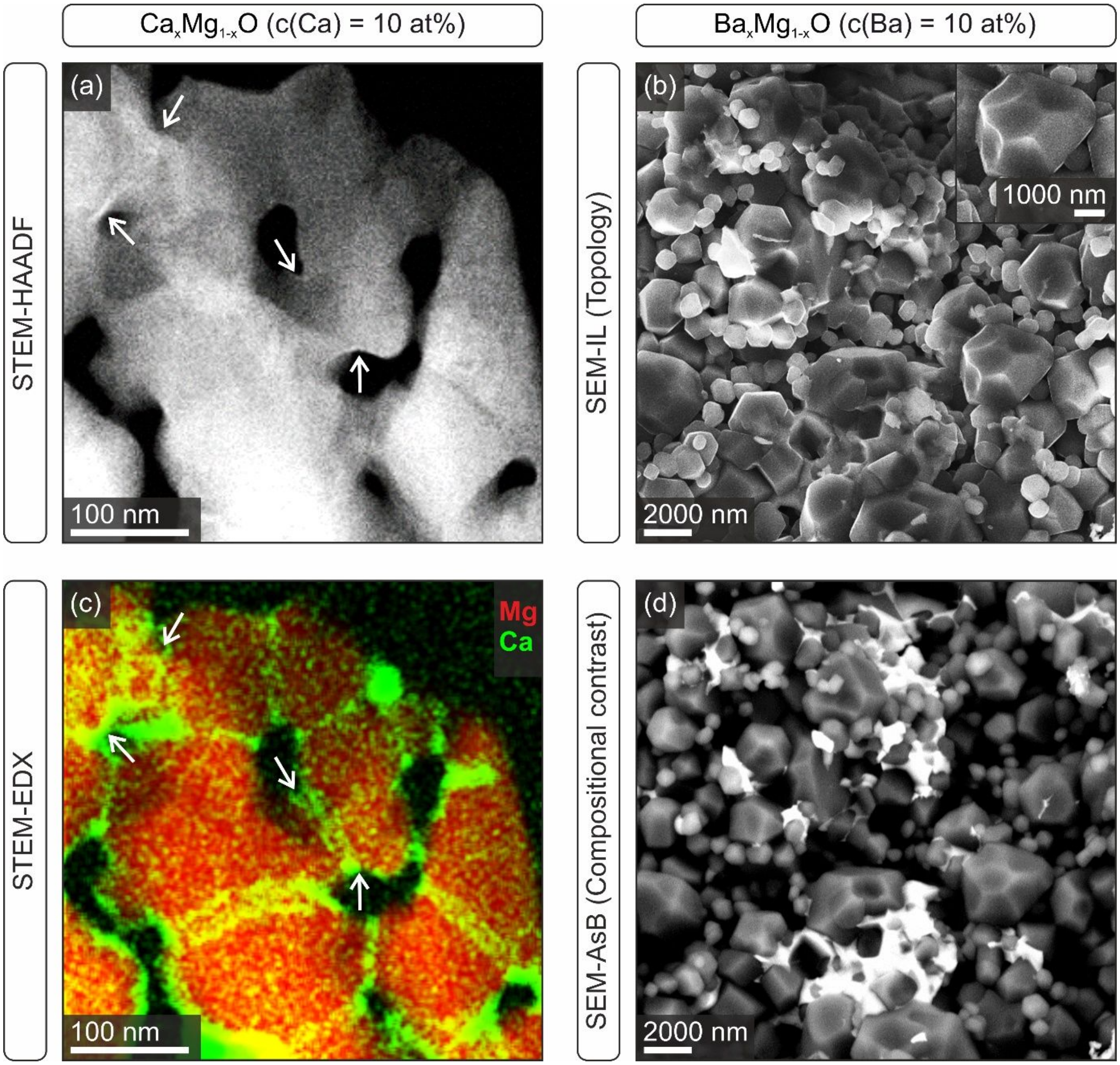

Figure S5. Electron microscopy analysis revealing the result of Ca- and Ba-segregation in $Ca_xMg_{1-x}O$ (*c*(Ca) = 10 at%, left a, c) and $Ba_xMg_{1-x}O$ (*c*(Ba) = 10 at%, right b, d) ceramics sintered at 1373 K. STEM-HAADF images (a, top left) provide information about the compositional contrast. White arrows indicate regions of enhanced contrast attributed to Ca-accumulation and confirmed by STEM-EDX intensity maps (c, bottom left). Secondary electron (b, top right) and backscattered electron SEM micrographs (d, bottom right) for ceramic fracture surface topology (IL-detector) and composition (AsB-detector) analysis on $Ba_xMg_{1-x}O$ (*c*(Ba) = 10 at%) are shown on the right.

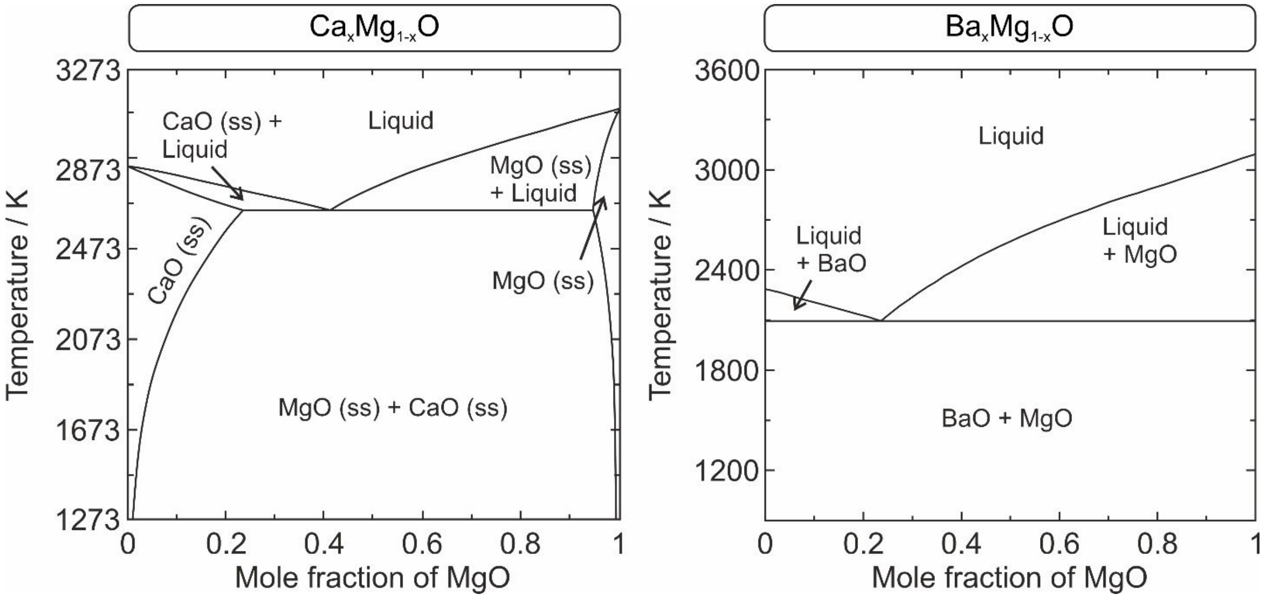

Figure S6. Equilibrium phase diagrams of MgO-CaO[1] (left) and MgO-BaO[2] (right) solid solutions.

Details about the approximation (Rule of mixture[3]) of mixed metal oxide densities:

A: Host material:

MgO $M(\text{MgO})$ = 40.30 g mol^{-1} $\rho(\text{MgO})$ = 3.58 g cm^{-3}

B: Impurity oxides:

BaO: $M(\text{BaO})$ = 153.33 g mol^{-1} $\rho(\text{BaO})$ = 5.70 g cm^{-3}

CaO $M(\text{CaO})$ = 56.08 g mol^{-1} $\rho(\text{CaO})$ = 3.34 g cm^{-3}

Conversion of atomic (x) into weight (w) percent:

$$w_A[\%] = \frac{M_A \cdot x_A}{M_A \cdot x_A + M_B \cdot x_B} \cdot 100$$

$$w_B[\%] = \frac{M_B \cdot x_B}{M_A \cdot x_A + M_B \cdot x_B} \cdot 100$$

Volume fraction f:

$$f[-] = \frac{w_B}{w_B + (100 - w_B) \cdot \frac{\rho_B}{\rho_A}}$$

$Me_xMg_{1-x}O$ composite density:

$$\rho_{Me_xMg_{1-x}O}[g \cdot cm^{-3}] = f \cdot \rho_B + (1 - f) \cdot \rho_A$$

Example for c(Ba) = 10 at%:

Assuming a complete conversion of admixed impurity ions into corresponding oxides as shown in the phase diagrams (Figure S6):

$$w_B[\%] = \frac{M_B \cdot x_B}{M_A \cdot x_A + M_B \cdot x_B} \cdot 100 = 29.7\ \%$$

$$f[-] = \frac{w_B}{w_B + (100 - w_B) \cdot \frac{\rho_B}{\rho_A}} = 0.21$$

$$\rho_{Ba_xMg_{1-x}O\ (c(Ba)\ =\ 10\ at\%)}[g \cdot cm^{-3}] = f \cdot \rho_B + (1 - f) \cdot \rho_A = 4.03\ g \cdot cm^{-3}$$

Details about the approximation of surface coverage (Figure 5):

MgO nanocubes (see also [4]):

Lattice constant (MgO): $a = 4.21 \cdot 10^{-10}$ m

Edge length (MgO cube): $l_E = 5 \cdot 10^{-9}$ m; $2 \cdot 10^{-8}$ m; $1 \cdot 10^{-7}$ m; $3 \cdot 10^{-7}$ m

Edge ions: $n_E = l_E \cdot (0.5 \cdot a)^{-1}$

Plane ions: $n_P = n_E^2$

Surface ions: $n_S = 6 \cdot (n_P - 4) - 12 \cdot (n_E - 2) + 8$

Explanation: Six planes without corners – overlapping edges + corner ions

Total ions: $n_T = n_E^3$

Surface Mg^{2+}-ions: $n_{Mg^{2+}\ (surf.)} = 0.5 \cdot n_S$

Total Mg^{2+}-ions: $n_{Mg^{2+}\ (tot.)} = 0.5 \cdot n_T$

Coverage:

Assumption: i) complete segregation of substitutional impurities

ii) perfect wetting of segregate layers

Impurity concentration: $c_{Me^{2+}}$ [at%]

Substitutional Me^{2+}-ions: $n_{Me^{2+}\ (subst.)} = n_{Mg^{2+}\ (tot.)} \cdot c_{Me^{2+}} \cdot 100^{-1}$

Monolayers (MLs): $MLs = n_{Mg^{2+}\ (surf.)} \cdot \left(n_{Me^{2+}\ (subst.)}\right)^{-1}$

References

(1) Wu, P.; Eriksson, G.; Pelton, A. D. Critical Evaluation and Optimization of the Thermodynamic Properties and Phase Diagrams of the CaO-FeO, CaO-MgO, CaO-MnO, FeO-MgO, FeO-MnO, and MgO-MnO Systems. *J. Am. Ceram. Soc.* **1993**, *76*, 2065–2075.

(2) Zhang, R.; Taskinen, P. A Thermodynamic Assessment of the BaO-MgO, BaO-CaO, BaO-Al_2O_3 and BaO-SiO_2 Systems. *Technical report - Science and Technology*, Aalto University, Helsinki, Finland, **2014**.

(3) Chawla, K. K. *Composite Materials: Science and Engineering*; Springer: New York, NY, **2013**.

(4) Thomele, D.; Gheisi, A. R.; Niedermaier, M.; Elsässer, M. S.; Bernardi, J.; Grönbeck, H.; Diwald, O. Thin Water Films and Particle Morphology Evolution in Nanocrystalline MgO. *J. Am. Ceram. Soc.* **2018**, 101, 4994–5003.
